# A cost–benefit analysis framework for preventive health interventions to aid decision-making in Australian governments

**DOI:** 10.1186/s12961-021-00796-w

**Published:** 2021-12-19

**Authors:** Jaithri Ananthapavan, Marj Moodie, Andrew Milat, Lennert Veerman, Elizabeth Whittaker, Rob Carter

**Affiliations:** 1 Deakin Health Economics, School of Health and Social Development, Institute for Health Transformation, Faculty of Health, Deakin University, Geelong, Australia; 2 Global Obesity Centre, School of Health and Social Development, Institute for Health Transformation, Faculty of Health, Deakin University, Geelong, Australia; 3 NSW Ministry of Health, New South Wales, Australia; 4 School of Public Health, Faculty of Medicine and Health, University of Sydney, Sydney, Australia; 5 School of Medicine, Griffith University, Gold Coast, Australia

**Keywords:** Cost–benefit analysis, Health policy, Decision-making, Preventive health

## Abstract

**Background:**

Australian governments are increasingly mandating the use of cost–benefit analysis (CBA) to inform the efficient allocation of government resources. CBA is likely to be useful when evaluating preventive health interventions that are often cross-sectoral in nature and require Cabinet approval prior to implementation. This study outlines a CBA framework for the evaluation of preventive health interventions that balances the need for consistency with other agency guidelines whilst adhering to guidelines and conventions for health economic evaluations.

**Methods:**

We analysed CBA and other evaluation guidance documents published by Australian federal and New South Wales (NSW) government departments. Data extraction compared the recommendations made by different agencies and the impact on the analysis of preventive health interventions. The framework specifies a reference case and sensitivity analyses based on the following considerations: (1) applied economic evaluation theory; (2) consistency between CBA across different government departments; (3) the ease of moving from a CBA to a more conventional cost-effectiveness/cost-utility analysis framework often used for health interventions; (4) the practicalities of application; and (5) the needs of end users being both Cabinet decision-makers and health policy-makers.

**Results:**

Nine documents provided CBA or relevant economic evaluation guidance. There were differences in terminology and areas of agreement and disagreement between the guidelines. Disagreement between guidelines involved (1) the community included in the societal perspective; (2) the number of options that should be appraised in ex ante analyses; (3) the appropriate time horizon for interventions with longer economic lives; (4) the theoretical basis and value of the discount rate; (5) parameter values for variables such as the value of a statistical life; and (6) the summary measure for decision-making.

**Conclusions:**

This paper addresses some of the methodological challenges that have hindered the use of CBA in prevention by outlining a framework that is consistent with treasury department guidelines whilst considering the unique features of prevention policies. The effective use and implementation of a preventive health CBA framework is likely to require considerable investment of time and resources from state and federal government departments of health and treasury but has the potential to improve decision-making related to preventive health policies and programmes.

**Supplementary Information:**

The online version contains supplementary material available at 10.1186/s12961-021-00796-w.

## Background

To ensure efficient use of limited government resources, policy-makers need to consider the costs and benefits of proposed government action against key comparators. Cost–benefit analysis (CBA) is a type of economic evaluation where the costs and benefits of a proposed policy action are considered in monetary terms. Australian federal and various state government central agencies (e.g. treasuries and the Department of Premier and Cabinet) recommend the use of CBA to inform significant government actions and mandate the use of CBA for submissions to the Cabinet [1–6].

The use of a CBA framework for policy appraisal is likely to be particularly useful for Cabinet decision-making, for several reasons. Firstly, CBA is the most common tool for policy appraisal across various sectors, such as transport and the environment [7, 8] and given that the Cabinet consists of government ministers from diverse sectors, an evaluation framework that is familiar to Cabinet members may assist decision-making. Secondly, when allocating resources across sectors, trade-offs need to be made, and hence appraisals across sectors need to be consistent and comparable [9, 10]. CBA is considered the only economic analysis technique that allows the assessment and valid comparison of interventions across sectors [11]. Finally, the CBA framework facilitates the capture of multiple benefits in a consistent analytical framework and therefore allows evaluation of policies with intersectoral impacts [10].

Valid comparisons between policies presented to the Cabinet at different times is possible only if there is sufficient standardization of CBA evaluation methodology and decision rules for policy approval [10]. However, any framework must have the flexibility to capture the credentials of interventions across a range of sectors. For example, New South Wales (NSW) Treasury guidelines report that CBA should take a NSW community perspective [4]. This may be appropriate for most analyses, but health interventions that result in cost-shifting to federally funded health services could appear more favourable than if a national perspective were taken. Therefore, the desire for harmonization of methodologies needs to be balanced with the need for adequate flexibility to allow robust analyses of specific policies [10].

Effective implementation of preventive health policies often requires political support, collaboration and coordinated action from various government sectors in addition to the health sector [12–15]. The intersectoral nature of preventive health interventions also increases the likelihood that Cabinet approval is required prior to implementation [2]. Prevention interventions are also often “complex”, with various health impacts across the population and spillover effects into other sectors [16]. For example, active transport policies require action from the transport sector and produce health benefits by increasing physical activity levels of individuals and environmental benefits from reduced reliance on private vehicles [17]. These features result in the current methods for healthcare economic evaluation, largely developed based on the medical model of healthcare, posing methodological challenges when used to evaluate preventive health interventions and policies. The key methodological challenges relate to the difficulty in establishing the efficacy of interventions where randomized controlled studies are not feasible, the effects of interventions emerging many years into the future—beyond the duration of conventional studies, additional effects not readily captured by health-related quality of life measures and spillover effects on other sectors [18–21]. Cost-effectiveness analysis (CEA) and cost-utility analysis (CUA) are the techniques predominantly used in the health sector; however, they do not adequately capture all the relevant costs and benefits of prevention policies [18, 19]. Despite the acknowledgement that there are difficulties in evaluating preventive health interventions, there is little consensus, guidance and application of appropriate methods to address the above challenges [19]. However, the recognition of these issues has resulted in the broadening of the paradigm of health economics beyond the medical model and an increased interest in CBA methods [22–24].

There are no clearly established best practice methods for the development of economic evaluation guidelines [25]. A consensus-based approach involving a small group of experts was used by the First and Second Panel on Cost-Effectiveness in Health and Medicine. The panels produced reference case recommendations aiming to increase the comparability and quality of economic evaluations in the health sector [9, 26, 27]. However, given the broad application of economic evaluation in health and the normative nature of many recommendations, several of the recommendations have been disputed [28, 29]. Governments around the world frequently use economic evidence in decision-making related to public reimbursement of health technologies [30]. Updates to these guidelines have involved reflecting on their own experiences to identify the incremental changes required [31, 32]. There is little detail available on the process for the development of CBA guidelines by various Australian government departments. The federal CBA guidelines and associated guidance notes, published by the Office of Best Practice Regulation (OBPR), do not specify the process for guideline development [33]. These guidelines provide high-level guidance on the conduct of CBA and specify a limited number of parameter values. They direct readers to CBA manuals for further information [33]. There is a trickle-down effect from these federal documents, with state treasury CBA guidelines being based on federal guidelines, and state line agency guidelines referring to their state treasury guidance [4].

Australian government decision-making involves a complex arrangement of shared powers between the federal and state governments. Public health is largely a shared responsibility between state and federal governments, hospitals are mainly funded by state governments and the federal government is responsible for primary care and the provision of pharmaceuticals [34]. This means that a CBA framework for preventive health interventions would need to be consistent with state treasury guidelines and designed to meet the needs of state cabinet decision-making, whilst also being comparable to other health sector economic evaluations. The latter are mostly evaluations undertaken for pharmaceuticals and health technology assessments that mainly use a CUA framework [31, 35].

Various government line agencies have developed sector-specific CBA guidance that use state treasury guidelines as a framework but provide specific practical advice tailored to sector-specific projects [8, 36–38]. The need for specific CBA guidelines for preventive health interventions arises from the several unique features of preventive health initiatives. Of particular concern when defining a CBA framework are issues with measuring and valuing effects across various sectors and the considerable time lag before intervention effects are realized [19, 39]. There is also a need for economic analyses to move easily from a CBA framework to a CEA/CUA framework so that preventive health interventions are comparable with other evaluations in the health sector.

The aim of this study is to develop a CBA framework for preventive health decision-making by Australian federal, state and territory governments, which balances the need for consistency with the requirements of central government agencies and other line agency guidelines and the guidelines and conventions of traditional health economic evaluations, whilst considering the impact of these specifications on the evaluation of preventive health interventions. A specified framework that defines a “reference case” with a core set of methods, parameter values and reporting of results can be used for the purposes of comparison [26], and alternative sensitivity analyses with justified variations from the reference case could allow the required flexibility.

## Methods

Given that preventive health decision-making is largely under the jurisdiction of state governments, the NSW State Government was used as an example for the development of the preventive health CBA framework. The NSW Government has been a leader in the use of economic evidence in decision-making and was the first state government in Australia to mandate the use of CBA, initially for new capital projects, and more recently with increased focus on using economic evidence in decision-making across all departments [10, 40, 41]. Within the NSW Government context, preventive health interventions that require new funding or impact various sectors often require Cabinet approval. This process involves the submission of a business case (which includes a CBA) to the NSW Treasury, who then informs the Cabinet Committee and the Cabinet Standing Committee on Expenditure Review [42]. This committee is chaired by the NSW Treasurer and consists of senior members from the Department of Premier and Cabinet and NSW Treasury [43].

### Document identification

The CBA framework for preventive health interventions is based on Australian federal and NSW Government department guidelines on economic evaluation and CBA. The included government departments are listed in Table 1. The government departments were selected based on their authority over economic evaluations for decision-making (e.g. federal and state central agencies) and the likelihood that the department had developed sector-specific CBA guidance that incorporates health impacts (e.g. Transport for NSW routinely uses CBA for the appraisal of policy proposals and includes health impacts in evaluations). The websites of these government departments were searched in January 2018 and again in August 2020 to identify any updates to the documents identified in the initial search. The search function in each of these websites was used with the following terms: “cost–benefit analysis”, “CBA”, “cost–benefit”, “benefit–cost”, “economic evaluation”, “guidelines”, “guidance” and “manual”. The titles and the short text below the first 10 pages of search results were reviewed to identify the most current CBA or other economic evaluation guidance documents and related documents reporting methods or values for use in economic evaluation (e.g. circulars related to the social discount rate).

**Table 1:** Australian federal and NSW government departments and agencies included in the website search

Federal government	Department of Prime Minister and Cabinet Office of Best Practice Regulation
	The Treasury
	Department of Finance
	Department of Health—the Pharmaceutical Benefits Advisory Committee
	Productivity Commission
	Infrastructure Australia
NSW government central agencies	NSW Treasury
	NSW Department of Premier and Cabinet
	Department of Finance, Services and Innovation (now part of the Department of Customer Service)
NSW government line agencies	NSW Ministry of Health Health Infrastructure Centre for Epidemiology and Evidence
	Transport for NSW
	Infrastructure NSW
	NSW Department of Planning, Industry and Environment

The research team conducted in-depth interviews and focus groups with participants from the NSW Ministry of Health and NSW Treasury in June–August 2018 to identify resource allocation decision-making processes for preventive health interventions in the NSW government [unpublished data]. Participants were asked to identify relevant documents that may assist in the development of a CBA framework for preventive health interventions. These documents complemented the website search.

### Data extraction and analysis

Data extraction focused on the key components of CBA and economic evaluations more generally. Data extraction involved JA initially extracting data on five documents and then presenting the initial data categories and results to RC and MM. The data extraction template was refined and used by JA to complete data extraction. Following data extraction, an analytical review was undertaken by JA to ascertain the similarities and differences between the recommended CBA methodology across the different departments and agencies, specifically those that impact economic analyses of preventive health policies and interventions. The assessment of CBA components that have relatively good agreement, poor agreement and flexibility in application involved a deliberative process with all authors until consensus was reached.

### Development of a reference case for preventive health interventions

The specifications of the reference case were based on the following considerations: (1) applied economic evaluation theory; (2) consistency between CBA across different NSW government departments; (3) the ease of moving from a CBA to a more conventional CEA/CUA framework used for health interventions, including consistency of decision context; (4) the practicalities of application by busy bureaucrats; and (5) the needs of end users, being both the Cabinet and decision-makers within health departments [9, 26, 27, 44].

When considering applied economic evaluation theory in relation to a CBA framework for preventive health, there is no authoritative textbook. The reference case draws from the handbook on CBA published by the Australian government [45] and the textbook that outlines the recommendations made by the Second Panel on Cost-Effectiveness in Health and Medicine [46], supplemented by key academic references related to specific topics. The impact of the various methodological specifications on the credentials of preventive health interventions are highlighted and used to inform the reference case and the recommended sensitivity analyses. Given that the NSW Treasury CBA guidance is the primary guidance document for CBA within the NSW government, when there was a conflict between guidelines, NSW Treasury guidance was used predominantly as the basis of the reference case.

Despite CBA being closely aligned to orthodox welfarist theoretical foundations where individual utility is the “maximand” (the thing to be maximized), this framework avoids the debate related to the appropriate normative foundation [welfarism or extra-welfarism (where health is the maximand)] for preventive health economic evaluations. We use the decision-making approach, [44, 47], which allows the development of a framework that is theoretically meaningful whilst flexible enough to broaden the concept of benefit (to capture the range of impacts relevant to preventive health decision-making [44]. This approach acknowledges that a range of applied economic techniques are appropriate and complementary as long as they meet the needs of the decision-maker [26, 44, 47]. See Additional file 1 for the definition of key terms.

## Results

The titles and short descriptions of 5234 website documents were screened, and an additional six documents were identified by NSW Health and Treasury staff (document search flowchart reported in Additional file 2). Full-text review was completed for 39 government documents (26 federal government and 13 NSW state government documents). Nine documents provided CBA or relevant economic evaluation guidance [4, 7, 8, 31, 33, 37, 45, 48, 49]. An overview of these nine guidance documents is reported in Table 2. Details of the perspective, comparator, specifications of the options for appraisal, time horizon and discount rate reported in these guidance documents are provided in Table 3. Table 4 provides an overview of guidance related to the costs and benefits that should be included or excluded, valuation of non-market benefits, key decision rules, sensitivity analyses and other key considerations in decision-making. Data relevant to CBAs extracted from the other reviewed government documents (*n* = 30) are reported in Additional file 3. An assessment of the CBA components that have relatively good agreement, poor agreement and areas where there is flexibility in application is provided in Table 5. Recommendations for the Preventive Health CBA Framework (hereafter referred to as the Framework) are outlined in Table 6.

**Table 2:** Overview of the key economic evaluation guidance documents

Jurisdiction	Department	Document title	Aims of document and overview of CBA guidance
Federal	PM&C (OBPR)	Cost–benefit analysis guidance note (2020) [33]	To provide Australian government and COAG policy-makers guidance on the use of CBA for policy proposals To promote comparability and consistency in decision-making and help maximize net benefits to society It provides an introduction to CBA for regulatory proposals, with readers referred to the *Handbook of Cost–Benefit Analysis* [45] for further details
Federal	Finance and Administration	Handbook of Cost–Benefit Analysis January (2006) [45]	To provide Australian government agencies guidance on the use of CBA for economic appraisal and decision-making and to provide details on the process of conducting CBA It provides no guidance on when CBA should be used
Federal	Infrastructure Australia	Assessment framework: for initiatives and projects to be included in the Infrastructure Priority List (2018) [48]	To provide guidance on the assessment framework used by Infrastructure Australia to consider projects for inclusion in the Infrastructure Priority List A detailed CBA is a central component of the business case
Federal	Health	Guidelines for preparing a submission to the Pharmaceutical Benefits Advisory Committee (2016) [31]	To ensure consistent, standardized submissions for consideration by the PBAC for the listing of new medicines on the PBS To ensure comparability of submissions to facilitate consistency in decision-making CUA rather than CBA is the preferred approach outlined in the guidance
NSW	Treasury	NSW Government Guide to Cost–Benefit Analysis (2017) [4]	To promote a consistent approach to appraisal and evaluation of publicly funded initiatives across the NSW government It advises that CBA guidelines by line agencies that are consistent with the framework and principles of this guide can be used for sector-specific evaluations It recommends that line agencies integrate CBA into decision-making processes
NSW	Transport	Transport for NSW Cost–Benefit Analysis Guide (2019) [8]	To outline the principles, concepts, methodology and procedures for conducting CBA for NSW Transport initiatives CBA is the preferred evaluation method and the key tool that is required both for compliance and to promote value-for-money government decision-making The guide outlines a consistent and best practice framework for NSW Transport evaluations. However, it emphasizes that it does not enforce compliance The guidance is aligned to NSW Treasury CBA guidance [4] and should be read in conjunction with federal government transport assessment guidelines
NSW	Planning, Industry and Environment	Guidelines for using cost–benefit analysis to assess coastal management options (2018) [7]	To provide specific guidance for LGA CBA related to coastal management It states that CBA is not a good use of council resources for all projects and should be used when making complex and high-risk decisions
NSW	Health (Health Infrastructure)	Guide to Cost–Benefit Analysis of Health Capital Projects (2018) [37]	To provide the principles, concepts and methodology to evaluate NSW Health capital projects To ensure that a robust analysis of the costs and benefits are considered in decision-making To provide a consistent and standardized approach to facilitate comparisons across capital projects The guidance is supplementary to the NSW Treasury CBA guidance [4]
NSW	Health (Centre for Epidemiology and Evidence)	Commissioning Economic Evaluations: A Guide (2017) [49]	This is not a guidance document on CBA, but aims to assist NSW Health staff commission economic evaluations, particularly related to population health programmes The document focuses on ex-post evaluations and states that the primary aim of economic evaluation is to inform the investment decision

*CBA* cost–benefit analysis, *COAG* Council of Australian Governments, *CUA* cost-utility analysis, *LGA* local government area, *NSW* New South Wales, *OBPR* Office of Best Practice Regulation, *PBAC* Pharmaceutical Benefits Advisory Committee, *PBS* Pharmaceutical Benefits Scheme, *PM&C* Prime Minister and Cabinet

**Table 3:** Guidance related to perspective, comparator/base case, options for appraisal, time horizon and discount rate

Document title	Perspective and referent group	Comparator/base case	Options for appraisal, (number and specifications)	Time horizon	Discount rate theoretical foundation and value for primary analysis (range for sensitivity analyses)
Cost–benefit analysis guidance note (2020) [33]	Societal: impacts on all Australian residents	Business as usual, do nothing	≥ 3; 1 non-regulatory option	Life of policy; 10 years for regulatory costs [85]	Opportunity cost of capital: 7% (3% and 10%)
Handbook of Cost–Benefit Analysis January (2006) [45]	Societal	Business as usual, do nothing	Not specified	Life of policy	Opportunity cost of capital: no values given
Assessment framework: for initiatives and projects to be included in the Infrastructure Priority List (2018) [48]	Societal: Australian community	Do minimum	Step 1: high-level MCA on long list of options. Step 2: rapid CBA on short list and more detailed MCA. Step 3: detailed CBA on final short list (≥ 2 options)	Life of asset. Duration needs to be justified	Theory not stated: 7% (4% and 10%)
Guidelines for preparing a submission to the Pharmaceutical Benefits Advisory Committee (2016) [31]	Healthcare system Optional: Societal	Existing technology/clinical practice replaced by the proposed medicine	Proposed new medicine	Duration to capture all important differences in costs and benefits between intervention and comparator	Theory not stated: 5% (0% & 3.5%)
NSW Government Guide to Cost–Benefit Analysis (2017) [4]	Societal: NSW community	Business as usual, no policy change, status quo	Full range of realistic options; variations in scale and scope, targeting supply and demand, alternative implementation plans	Life of policy/asset; 20–30 years	Opportunity cost of capital: 7% (3% and 10%)
Transport for NSW Cost–Benefit Analysis Guide (2019) [8]	Cites NSW Treasury guidance [4]: Societal: NSW community	Business as usual, do minimum	≥ 3 for large projects and ≥ 2 for smaller projects	Life of asset. Cites NSW Treasury guidance [4] (30 years). No longer than 50 years	Social rate of time preference: cites NSW Treasury guidance [4]: 7% (3% and 10%)
Guidelines for using cost–benefit analysis to assess coastal management options (2018) [7]	Societal: LGA community	Continuation of current management, business as usual	Number not specified. Options should be discrete and not reliant on other options. Variations in options treated as individual options	Life of project. Cites NSW Treasury guidance [4] (30 years). Longer time horizons can be adopted (e.g. 50 years)	Theory not stated: cites NSW Treasury guidance [4]; however, lower value differs: 7% (4% and 10%)
Guide to Cost–Benefit Analysis of Health Capital Projects (2018) [37]	Cites NSW Treasury guidance [4]: Societal: NSW community	Status quo, keep safe and operating	Step 1: high-level CBA on long list of options. Stage 2: detailed CBA on short list (≥ 2 options)	Practical asset life; 20–30 years	Cites NSW Treasury guidance [4]: 7% (3% and 10%)
Commissioning Economic Evaluations: A Guide (2017) [49]	Health sector Optional: whole of government, societal	Current practice	Not specified (focus of guidance on ex-post evaluations)	Length of time over which outcomes are likely to accrue	Reports recommended rates vary by jurisdiction. Cites NSW Treasury guidance [4]; however, a recommended rate not stated

*CBA* cost–benefit analysis, *LGA* local government area, *MCA* multi-criteria analysis, *NSW* New South Wales, *OBPR* Office of Best Practice Regulation, *PM&C* Prime Minister and Cabinet

**Table 4:** Guidance related to quantification and valuation of impacts, decision rules, sensitivity analyses and other considerations

Document title	Costs and benefits	Valuation of non-market benefits	Key decision rules	Sensitivity/uncertainty analyses	Other considerations
Cost–benefit analysis guidance note (2020) [33]	Productivity identified as first-round impacts Exclude: second-round impacts (e.g. land use)	Quantify if able, otherwise qualitative assessment RP preferred, SP, benefit transfer VSL (A$ 2021) = 5.1 M VSLY (A$ 2021) = $222,000 [74]	NPV	Worst/best-case, one-way, Monte Carlo methods	Distributional impacts
Handbook of Cost–Benefit Analysis January (2006) [45]	Productivity identified as a benefit of health interventions Exclude: WEB including employment multipliers, second-round impacts	Quantify if able. Non quantified impacts are called “intangibles” and should be described qualitatively RP, SP, benefit transfer	NPV. Cautious use of BCR which can be biased towards small projects	Worst case, one-way, Monte Carlo methods for complex cases	Distributional impacts
Assessment framework: for initiatives and projects to be included in the Infrastructure Priority List (2018) [48]	Productivity identified as a benefit Categorized into impacts on users and producers and external impacts to broader community Where appropriate include: WEB, second-round land-use benefits	Quantify if able, otherwise qualitative assessment RP, SP, replacement cost method, benefit transfer	NPV, BCR Others: NPV per dollar of capital investment, first year rate of return	Time horizon: 30 and 50 years Include/exclude WEB Best-case scenario: −20% costs and + 20% benefits/upside adjustment for 4–5 key variables Worst-case scenario: + 20% costs and − 20% benefits/downside adjustment for 4–5 key variables Monte Carlo—probability distribution of project costs with 50% and 90% probability that the cost won’t be exceeded	WEB, land-use impacts, productivity, urban regeneration, local equity and distributional impacts
Guidelines for preparing a submission to the Pharmaceutical Benefits Advisory Committee (2016) [31]	Include: all health sector impacts Exclude: non-health sector costs, productivity impacts in primary analysis	Recommends population preference weights for health state utility calculation	ICER; $ per QALY gained	Requires extensive one-way, multi-way, Monte Carlo methods	Equity of access to medicine
NSW Government Guide to Cost–Benefit Analysis (2017) [4]	Productivity is a direct impact on individuals and an indirect impact on employers Include: first-round impacts (direct and indirect) Exclude: second-round impacts (e.g. WEB, land value uplift)	Quantify if able, otherwise qualitative assessment Valuation based on individuals/firms that experience outcomes RP preferred, SP, benefit transfer	NPV, BCR. BCR used when there are differences in ranking between NPV and BCR	All key values and assumptions using one-way, scenario, best/worst-case, Monte Carlo methods WEB and land uplift can be included in best-case scenario	Distributional impacts
Transport for NSW Cost–Benefit Analysis Guide (2019) [8]	Impacts on the user, social, government, dis-benefits, other impacts (WEB, land value uplift, option value and nonuse value, improvements to place) Exclude WEB in primary analysis, take care when using land uplift values	Quantify if able, otherwise qualitative assessment RP, SP VSL (A$ 2019) = 7.6 M [70]	NPV, BCR. BCR used when differences in ranking between NPV and BCR	Deterministic and probabilistic (Monte Carlo) methods	Distributional impacts
Guidelines for using cost–benefit analysis to assess coastal management options (2018) [7]	Include: Direct and indirect impacts and externalities (use and nonuse values) Exclude: second-round impacts	Quantify if able, otherwise qualitative assessment RP, SP, benefit transfer	NPV, BCR. No advice on the more appropriate option when ranking differs. Reports decision-making should be based on both criteria	All key values and assumptions tested using Monte Carlo methods	Distributional impacts across public and private sector stakeholders Second-round impacts to local businesses, employment, income and social cohesiveness impacts
Guide to Cost–Benefit Analysis of Health Capital Projects (2018) [37]	Productivity direct impact on individuals and indirect impact on employers Include: first-round impacts (direct and indirect) Exclude: second-round impacts (e.g. WEB, land value uplift)	Quantify if able, otherwise qualitative assessment RP preferred, SP, benefit transfer Methods for valuation of health benefits by service stream provided in the CBA toolkit [15]. DALYs valued using VSLY VSL (A$ 2018/19) = $4.5 M VSLY (A$ 2018/19) = $195,000	NPV, BCR. BCR used when differences in ranking between NPV and BCR	All key values and assumptions tested using one-way, scenario, best/worst-case, Monte Carlo methods	Distributional impacts
Commissioning Economic Evaluations: A Guide (2017) [49]	Impacts measured and quantified depend on the perspective of the evaluation. Include: direct (provider costs), indirect (patient, family, including absenteeism), cost offsets (health sector cost savings), non-healthcare cost offsets (cost savings to other sectors)	Health valued using QALY and DALY. Reports there are equity concerns for using WTP values to monetize health benefits, as WTP is associated with ability to pay	ICER Reports PBAC threshold based on previous decisions (A$ 37,000–69,000 per life-year gained)	One-way, scenario, Monte Carlo methods	Distributional impacts

*A$* Australian dollars, *BCR* benefit–cost ratio, *CBA* cost–benefit analysis, *DALY* disability-adjusted life-year, *ICER* incremental cost-effectiveness ratio, *NPV* net present value, *NSW* New South Wales, *PBAC* Pharmaceutical Benefits Advisory Committee, *QALY* quality-adjusted life-year, *RP* revealed preference, *SP* stated preference, *VSL* value of a statistical life, *VSLY* value of a statistical life-year, *WEB* wider economic benefits, *WTP* willingness to pay

**Table 5:** CBA components that have relatively good agreement, poor agreement and flexibility in application

CBA component	Areas of agreement	Areas of disagreement	Areas of flexibility
When to conduct a CBA	Commensurate with the scale of investment Required for investments over A$ 10 million	CBA not recommended for primary analysis by PBAC	Type of analysis that is appropriate for investments less than A$ 10 million
Perspective and referent group	Societal perspective for CBA	The referent group or standing varies from communities within LGAs, the state jurisdiction and whole of Australia Health perspective recommended by PBAC using CUA	
Comparator/base case	Well-defined status quo. Avoidance of “straw man” comparator	Terminology (base case versus comparator)	
Options for appraisal	A range of realistic options should be included	The number of options included	The nature of options included
Time horizon	Principle that the time horizon should be the economic life of the project Longer time horizons are associated with increased uncertainty	Appropriate time horizons for various interventions	
Social discount rate		Theoretical basis for social discount rate The rate to be used in primary and sensitivity analyses	
Costs and benefits	Second-round impacts excluded in primary analyses	The appropriate value for the VSL and VSLY	Technique for estimating non-market impacts Inclusion of productivity impacts
Decision rules	NPV and BCR should be reported	The preferred outcome measure (NPV or BCR) when these measures give varied results	
Sensitivity analyses	Extensive uncertainty analysis should be undertaken to test the impact of key assumptions and variables	Terminology (uncertainty analysis, risk analysis, sensitivity analysis)	Type of analyses: one-way, scenario, best/worst-case, Monte-Carlo simulations
Distributional impacts and other considerations	Distributional impacts should not be incorporated into technical results Distributional impacts should be considered by decision-makers		Method of undertaking distributional analysis
Reporting	All critical assumptions and input parameters should be documented with supporting evidence	Reporting standardized but varied across guidelines	

*A$* Australian dollars, *BCR* benefit–cost ratio, *CBA* cost–benefit analysis, *CUA* cost-utility analysis, *LGA* local government area, *NPV* net present value, *NSW* New South Wales, *PBAC* Pharmaceutical Benefits Advisory Committee, *VSL* value of a statistical life, *VSLY* value of a statistical life-year

**Table 6:** Recommendations for the Preventive Health CBA Framework and associated rationale

CBA component	Key recommendation [rationale]
When to conduct a CBA	Investments over A$ 10 million [ii; iv] For investments less than A$ 10 million, CBA should be commensurate with the size of investment and built into the decision-making process [iv; v]
Perspective and referent group	Primary analysis using a societal perspective [i—clear concept of benefit; ii] Health sector perspective for additional analyses [iii] State-based community referent group [iv; v]
Comparator/base case	Defined as the status quo [i—comparative analysis; ii; iii] Comparator referred to as the base case [ii]
Options for appraisal	Number of options commensurate with the size of the investment [i—comparative analysis; iv] Options underpinned by government health strategy [v] MCA to establish short tractable list of options for detailed CBA. The MCA should use criteria commonly used in preventive health decision-making [ii; iv]
Time horizon	Up to 30 years based on the nature of the intervention [ii] and lifetime/100 years if 30 years unlikely to capture all important impacts [i—capture the full economic life of project; iii]. The most appropriate time horizon with justification should be used for the primary analysis, with the other used in sensitivity analyses
Social discount rate	Base case 3% [i; iii] Sensitivity analyses using 0%, 5%, 7% and 10% [ii; v]
Costs and benefits	All impacts consistent with societal perspective identified, including healthcare costs borne by all payers (including federal government) [i—clear concept of benefit, opportunity cost; iii; iv]. Important impacts measured and valued [iv]. This should be commensurate with the size of investment Report proportion of healthcare cost/cost savings that will accrue to state government compared to other funders [ii; v] Develop logic models to identify potential impacts across all sectors [i—opportunity cost] Quantify and value significant impacts across all sectors and report these by sector [iii] Quantify health impacts using health-related quality-of-life measures (QALY or DALY) [iii] Value DALY/QALY using VSLY (A$ 303,531, in 2017 values). This value should be consistent across all CBAs across all jurisdictions [ii] Productivity impacts excluded in primary analysis [iii; iv] Sensitivity analyses: VSLY values: A$ 315,732 and A$ 88,136 (in 2017 values). A range of values should be tested when using a health sector perspective VSLY to value life-years (LY) rather than DALYs/QALYs Include indirect productivity impacts on employers using the FCA and gender-free wage rates
Decision rules	NPV and BCR. BCR basis of decision-making when intervention rankings differ between the two [ii] All impacts resulting from an intervention should be accounted for on the benefits side of the equation when calculating BCR [ii]
Sensitivity analyses	One-way, scenario and probabilistic sensitivity analyses undertaken to assess the variability in the results [ii; iii; v] All input parameters and assumptions should be documented with mean values, distributions and the sources [v] Avoid the terms “uncertainty analysis” and “risk analysis”
Distributional impacts and other considerations	Primary analyses should not include equity or other impacts in the technical CBA results [ii] Full description of equity and distributional impacts with quantification of impacts across subgroups where appropriate [v] Full description of other important considerations related to preventive health interventions reported qualitatively and quantitatively [v]
Reporting	Full description of options for appraisal and the assumptions and inputs used in the analysis [v] Results disaggregated by sector and reported by method of measurement [iii; v] Full documentation and interpretation of primary analysis, sensitivity analyses and distributional impacts [v]

Rationales: (i) economic theory, (ii) consistency of CBA across different state government departments, (iii) consistency with other health intervention evaluations and the ease of moving from a CBA to a more conventional CEA/CUA framework used for health interventions, (iv) the practicalities of application by busy government bureaucrats, and (v) the needs of the end user

*A$* Australian dollars, *BCR* benefit–cost ratio, *CBA* cost–benefit analysis, *CUA* cost-utility analysis, *DALY* disability-adjusted life-year, *FCA* friction cost approach, *MCA* multi-criteria analysis, *NPV* net present value, *NSW* New South Wales, *PBAC* Pharmaceutical Benefits Advisory Committee, *QALY* quality-adjusted life-year, *VSL* value of a statistical life, *VSLY* value of a statistical life-year

### When should CBA be used in the health sector?

The CBA/economic evaluation guidance documents generally state that the purpose of CBA is to assist decision-makers make value-for-money decisions using a consistent decision-making approach. The guidelines make recommendations on when to complete a CBA based on various factors including the size of the investment [4, 37, 49]; the nature of the investment, with regulatory interventions requiring a CBA [4, 33]; the policy decision-maker, with proposals to Cabinet requiring a CBA [33]; and the level of complexity and risk [4, 49]. There is wide agreement that the scale of the analysis should be commensurate with the scale of the project [4, 37, 49], with some guidelines reporting that the viability of smaller projects could be threatened by the cost of conducting an ex-ante CBA [7, 45].

*Framework recommendation* To maintain consistency with Treasury guidance [4], the Framework recommends an ex-ante CBA for preventive health interventions with investments over A$ 10 million. It is recommended that for investments less than A$ 10 million, ex-ante analyses commensurate with the size of investment are undertaken. Although the CBA should be consistent with the specifications of the Framework, the number of options evaluated can be reduced and quantification can be limited to the key parameters and main impacts of the intervention, with greater use of qualitative analysis of other inputs and impacts. This will ensure that economic evidence is built into the decision-making process for all preventive health interventions as recommended by Treasury [4], whilst ensuring that the associated costs are appropriate.

### What is the appropriate perspective/scope for the Preventive Health CBA Framework?

All CBA guidance documents [4, 7, 8, 33, 37, 45, 48] recommend using a “societal perspective” in CBA where the impacts on all stakeholders are incorporated into the evaluation. However, the recommended scope (also called the referent group, or the standing) of the analysis varies across guidance documents from communities in specific local government areas (LGAs) [7], the state [4, 8, 37], to all Australian residents [33, 48]. NSW Treasury [4] recommends a societal perspective that includes the NSW community (households, businesses, workers and/or governments) and is cited by NSW Health Infrastructure [37] and Transport for NSW [8] guidance. One of the key recommendations from the Second Panel on the Cost-Effectiveness in Health and Medicine is the consistent use of a societal perspective in addition to the healthcare payer perspective in all health-related economic evaluations [46]. Despite this change in focus, the Pharmaceutical Benefits Advisory Committee (PBAC) [31] continues to recommend a healthcare system perspective, with all impacts not related to health or the provision of healthcare excluded in the primary analysis, though additional analyses using a societal perspective can be included in the submission. Consistent with PBAC guidance, the NSW Health Centre for Epidemiology and Evidence [49] acknowledges that it would be ideal to adopt a societal perspective in economic evaluations, but reports a health sector perspective as the most commonly used. It suggests that alternative perspectives could be used for secondary analyses (whole-of-government, societal).

The complexity of healthcare provision and financing in Australia [50] is not acknowledged in the NSW Health Infrastructure guidance [37]. It recommends a societal perspective, with the NSW community as the referent group, whilst recommending that costs largely accrued by the federal government be included in analyses (e.g. non-admitted patient services are included in NSW Health Infrastructure CBA, but primary care, a key cost component of non-admitted patient services, is funded by the federal government).

*Framework recommendation* To maintain consistency with Treasury guidance [4], the Framework recommends a societal perspective. Defining the referent group is not straightforward because it is difficult to accurately disaggregate healthcare costs borne by state and federal governments. Cost-shifting to payers outside the state does not represent increased efficiency [50], and therefore the Framework recommends the inclusion of all healthcare costs regardless of whether they are borne by the state or federal government. However, to inform state government decision-making, the analyst should attempt to estimate the proportion of cost/cost savings accrued by the community within the state jurisdiction. To allow comparison with other health interventions, the conduct of additional analyses using a health sector perspective is recommended [46].

### What is the appropriate comparator for the Preventive Health CBA Framework?

Economic analysis involves a comparative assessment; however, the definition of the comparator depends on the question being addressed [46]. For government decision-making, the question generally is, “Is the state of the world with the programme better than the state of the world without the programme?” In line with this question, all guidelines define the comparator as the “status quo”. All guidelines emphasize the importance of defining an appropriate comparator and warn against the use of a “straw man” comparator that may not accurately capture the credentials of the proposed policy [4, 7, 31, 33, 37, 48]. There are differences in terminology used in CBA guidance and health sector guidance. CBA guidance [4, 7, 8, 33, 37, 45, 48] refers to the comparator as the “base case”, whilst the health-based guidelines (PBAC [31] and the NSW Health Centre for Epidemiology and Evidence [49]) use the term “comparator”. PBAC [31] uses the term “base case” to refer to the primary analysis.

*Framework recommendation* The Framework recommends a well-defined “status quo” or “current practice” comparator. To maintain consistency with other CBA, this should be referred to as the “base case”. However, the differences in typology should be considered when communicating results to various audiences.

### Defining the options for appraisal for the Preventive Health CBA Framework

When conducting an ex-ante CBA, treasury departments [4, 51–53] recommend a process whereby the full range of realistic options for achieving the aims of the proposed policy are developed. Different guidance documents report a variety of processes to refine and develop the final short list of options for detailed appraisal [8, 37, 48]. Some guidelines specify the number of options—usually at least two or three—required to be analysed, whilst some guidelines require specific options included (e.g. for regulatory impact statements, one non-regulatory option is required [33]).

*Framework recommendation* Given the time and expense involved in evaluating a long list of options, the principle that the CBA should be commensurate to the size of the investment can be used to limit the number of options evaluated for preventive health interventions [8]. To be consistent with Treasury guidance [4, 51–53] while still keeping the number of options tractable, it is recommended that health strategy documents underpin option selection [37]. A manageable list of options should be assessed, potentially using multi-criteria analysis (MCA) as recommended by Infrastructure Australia [48]. MCA can be used to quantitatively and qualitatively assess how well each of the options meets specific criteria. The results can then be used to guide decision-making on the shorter list of options for CBA appraisal. Undertaking MCA using criteria commonly used in preventive health decision-making (e.g. evidence of intervention effectiveness [54, 55]) will increase the tractability of this task.

### What is the appropriate time horizon for the Preventive Health CBA Framework?

Economic theory suggests that the appropriate time horizon is the economic life of the project and should be long enough to capture important differences in costs and benefits between options and the base case (comparator) [45, 46]. This is acknowledged by all the guidance documents; however, several acknowledge the uncertainty involved in forecasting over extended periods [4, 31, 33]. NSW Treasury [4] states that time horizons longer than 20–30 years need to be discussed with the Treasury department prior to line agencies conducting ex ante analyses. Based on Treasury [4] guidance, NSW Health Infrastructure [37] has set a time horizon of 20 years for all evaluations, while Transport for NSW [8] report that longer time horizons (up to 50 years) may be appropriate; however, the plausibility of data and assumptions are required to be verified with the Transport for NSW Evaluation and Assurance team. CBA guidelines from federal and other Australian state jurisdictions do not specify time horizon limits, but some make recommendations based on the estimated economic life of various assets [38, 45, 48, 52, 53].

The nature of preventive health intervention is that the time frame between intervention implementation and effect is much longer than that for many other health interventions [16] and policies in other sectors. Although transport interventions may result in benefits accruing many years into the future, the intervention benefits start accruing soon after intervention implementation. However, for example, chronic disease prevention interventions in children are likely to have minimal benefits until the cohort reaches middle age, with benefits increasing as the intervention population reaches advanced ages [56]. Therefore, CBA using shorter time horizons is likely to disproportionately disadvantage prevention initiatives. Time horizons over the lifetime of the population (using population-modelling techniques) have often been used to evaluate preventive health interventions using a CUA framework [57, 58]. The uncertainties associated with modelling behaviour change is routinely incorporated in sensitivity analyses using various assumptions related to the intervention effect decay over longer time horizons [57, 58].

*Framework recommendation* A time horizon that is appropriate to the proposed policy should be used and justified as the primary analysis. A time horizon of up to 30 years to maintain consistency with NSW Treasury [4] guidance, or a longer time horizon up to the lifetime of the population with appropriate consideration of the uncertainty in the projections, can be chosen for the primary analysis. If a time horizon up to 30 years is not used in the primary analysis, it should be included in sensitivity analyses.

### What is the appropriate social discount rate for the Preventive Health CBA Framework?

The social discount rate is used to express all costs and benefits in present-day prices. The theoretical basis of the discount rate remains a topic of debate with little consensus [19, 29, 59]. The two most common approaches for estimating the discount rate are based on the opportunity cost of capital and the social rate of time preference. The social rate of time preference can be estimated from the rate of return of long-term government bonds; however, the opportunity cost of capital cannot be measured directly, and empirical estimations are likely to be subject to measurement error [60]. NSW Treasury [4] reports the opportunity cost of capital incorporating non-diversifiable market risk as the theoretical basis for using a real discount rate of 7% for the primary analysis for CBAs undertaken across all sectors. It states that the discount rate should remain stable over the analysis period and that the impact of varying the discount rate should be tested in sensitivity analyses (using a rate of 3% and 10%). It further stipulates that project risk should be reflected in the quantification of the costs and benefits and not in the discount rate. This is largely consistent with the federal Department of Prime Minister and Cabinet (PM&C) [33] guidance. However, in specific PM&C guidance related to environmental impacts [33], a declining long-term discount rate is recommended, which drops gradually from 5.4% for periods of analysis over 30 years, to 3.7% for periods over 301 years. NSW line agencies (Health Infrastructure [37], Transport for NSW [8] and NSW Planning, Industry and Environment [7]) guidance documents cite NSW Treasury [4] guidance. However, there are some discrepancies, with Transport for NSW [8] reporting that the discount rate is based on the social rate of time preference rather than the opportunity cost of capital, and NSW Planning, Industry and Environment [7] recommending a rate of 4% in sensitivity analyses.

The PBAC [31] recommend a discount rate of 5% applied uniformly to costs and benefits and sensitivity analyses using 0% and 3.5%; no explanation of the theoretical basis of the chosen rates is provided. The NSW Health Centre for Epidemiology and Evidence [49] reports that one rationale for discounting is the social rate of time preference. It emphasizes that population health programme evaluations are particularly sensitive to the discount rate. Federally funded transport projects are required to conduct CBA using the rate recommended by Infrastructure Australia (7% for primary analysis and 4% and 10% for sensitivity analyses) [48]. The Transport for NSW [8] guidance states that for projects with long lives, the narrative around the results should highlight the appropriateness of lower discount rates.

Like the time horizon, the discount rate has a considerable impact on preventive health interventions, where the benefits are likely to occur several years into the future. Many academic preventive health studies have used a discount rate of 3% [57, 58], which is the rate recommended by the original and Second Panel on Cost-Effectiveness in Health and Medicine (based on the social rate of time preference) and WHO [27, 46, 61]. Several economists have argued that current discount rates are too high, and have advocated for rates as low as 1.5% [62–65]. The Council of Economic Advisers in the United States recently recommended lowering the social discount rate based on either the opportunity cost of capital or the social rate of time preference in light of recent empirical evidence and theoretical advances [60].

Although there is consistency between the rate recommended by federal and NSW Treasury CBA guidelines, it is argued that this consistency is the result of circular referencing and the use of a limited number of outdated references [66]. It is argued that the 7% discount rate recommended by the federal government and NSW Treasury does not reflect contemporary economic thinking or practice [64] and is considered too high for the appraisal of public sector projects in general, and specifically for health-related projects. The key components of the discount rate based on the opportunity cost of capital is the risk-free rate of return and the market risk premium [60, 66]. The risk-free rate of return, which can be estimated from the 10-year Commonwealth bond rate, has steadily dropped from 6.8% in 1989, when the 7% discount rate was first established in Australia, to 0.8% in 2017 [66]. The measurement of market risk premium is more complex, with various uncertainties, but has been estimated to be relatively stable [60, 67]. This indicates that the discount rate should have dropped by 6% over this time period [66]. NSW Treasury references the weighted average cost of capital (WACC) calculated by the Independent Pricing and Regulatory Tribunal to justify the 7% discount rate for primary analyses; however, current estimates of the WACC (July 2019) have been lowered to 4.7% [67].

The Victorian Department of Treasury and Finance recommends various discount rates across sectors, with investments in public health required to use a 4% discount rate, whereas other sector projects use 7% in economic analyses [52]. Queensland Treasury reports that the discount rate should be project-specific and needs to be determined in consultation between Treasury and the specific agency [53]. The PBAC, an Australian federal government agency [31], recommends a lower discount rate than the OBPR [33], also an Australian federal government agency. Several other countries also have higher discount rates recommended by their treasury and central departments and lower values recommended for health evaluations [59, 62]. It has been argued that, rather than a standard discount rate for all publicly funded projects, the discount rate should vary according to the project’s systematic risk (a project has low systematic risk if it yields consistent returns regardless of changes in the economy as a whole) [66]. Preventive health investments are likely to have low systematic risks, and a discount rate of 3.5% for projects with very low systematic risk has been recommended [66]. Several health economists assert that health is a unique commodity, and the consumption value of health grows as income increases over time, and therefore a discount rate lower than the social rate of time preference should be used for the evaluation of health interventions [29, 62, 68]. A final argument for lower rates relates to ethical considerations when evaluating interventions that have intergenerational impacts, where higher discount rates emphasize the benefits to current populations at the expense of younger people and future generations. This has been highlighted as an issue in the evaluation of vaccines [62, 63, 66, 69].

*Framework recommendation* A discount rate of 3% in the primary analysis is recommended on the basis of consistency with previous economic evaluations of preventive health interventions, WHO and the Second Panel on Cost-Effectiveness in Health and Medicine. Sensitivity analyses should use a range of values including 0%, 5%, 7% and 10% to highlight the impact of the discount rate on the CBA results.

### What impacts should be included in the Preventive Health CBA Framework?

All CBA guidelines agree that direct impacts (on those directly involved in the consumption or production of the intervention) and indirect impacts (on third parties not directly involved) should be included in the CBA, but flow-on or second-round impacts, such as income multipliers, excluded from the primary analyses. NSW Transport [8] and Infrastructure Australia [48] suggest that the inclusion of these second-round benefits may be appropriate in supplementary analyses. The PBAC [31] reports that only health sector costs and health benefits should be included in their analyses. Two guidance documents are supplemented with manuals with appropriate methods and values for commonly quantified impacts [31, 70].

Health and safety are often given as examples of impacts that are difficult to forecast [4, 37, 45]. NSW Treasury [4] specifically identifies the difficulties in estimating the effects of a programme where there may be multiple possible causes for the outcomes of interest, and impacts related to behaviour change. Both these issues are particularly relevant to preventive health interventions—however, no solutions are identified in the guidelines [4]. Behaviour change and issues of causal attribution in prevention are often addressed through validated epidemiological methods (e.g. using potential impact fractions, joint risk factor adjustments and other methods) to estimate the impact of the intervention on change in risk factors and final health outcomes of interest [71, 72]. However, these methods may be unfamiliar to central departments [unpublished data]. NSW Health Infrastructure [37] reports that the key difference between CBA in the health sector and other sectors is the complex interplay of factors that determine health in a community. These guidelines categorize health benefits according to impacts on healthcare service streams (emergency department, cancer services, etc.) with detailed methods for the quantification of benefits in disability-adjusted life-years (DALYs) [73]. These guidelines are focused on the provision of healthcare and have limited relevance to preventive health policies.

Revealed preference (RP), stated preference (SP) and benefit transfer are commonly reported techniques used to value non-market impacts. NSW Treasury [4] and PM&C [33] report that RP techniques are preferred to SP techniques, whilst other guidelines do not report a preference. Within the health sector, the monetary valuation of health is a contentious issue [49], but it is unavoidable when conducting a CBA or for decision-making when trade-offs between health and other outcomes are considered. The value of a statistical life (VSL) and the value of a statistical life-year (VSLY) are often recommended as appropriate methods to value health and safety benefits; however, the actual value of these parameters differs between guidelines. PM&C [74] provides an estimate of the VSL and VSLY based on a review of the international literature in 2008 [75]. The value is updated annually to account for inflation. A recent systematic review was undertaken to update the VSL and VSLY for Australian policy decision-making [76]. Based on the most contemporary Australian data, the authors recommended a VSL of A$ 7 million and a VSLY of A$ 303,531 (in 2017 values), with additional values for sensitivity analyses [76]. The value of a QALY used by PBAC in its decision-making is not explicit [77, 78]. NSW Health Infrastructure [37] uses the PM&C [74] VSLY value and recommends that it be used to assign a monetary value to a DALY. However, the two guidelines use different inflation indices, and therefore the recommended VSL/VSLY differs between the two guidelines. NSW Health Infrastructure reports that the VSLY is a value of a year of perfect health [37]. Although in practice this is a reasonable assumption, VSLY is estimated from VSL studies that do not assume the statistical life was in perfect health, and therefore could potentially underestimate the value of a DALY. The VSL used by Transport for NSW [70] is based on two SP studies and is 70% higher than the value used by NSW Health Infrastructure. The use of varied VSL and VSLY values in analyses across sectors and across jurisdictions may result in inconsistent decision-making, and therefore efforts to update and harmonize these values should be prioritized.

People who suffer from chronic illnesses earn less than their healthy counterparts, and ill health impacts productivity through absenteeism, presenteeism and premature death or retirement [79]. Productivity impacts on individuals are considered direct first-round impacts, and productivity impacts on employers are considered indirect first-round impacts [37]. NSW Health Infrastructure [37] and the NSW Health Centre for Epidemiology and Evidence [49] identify productivity benefits of reduced absenteeism as a potential benefit for patients and families and employers. NSW Health Infrastructure [37] reports that these impacts can be reported qualitatively. Transport for NSW [70] does not provide any guidance on the inclusion of productivity in CBA associated with the health impacts of transport interventions. The PBAC [31] (using a healthcare system perspective) recommends that the primary analysis exclude productivity impacts, but these may be included in supplementary analyses. It reports that there are equity implications in including productivity and potential issues with double-counting of benefits related to the valuation of health-related quality of life. Although evidence suggests that quality-adjusted life-years (QALYs) generally do not capture productivity impacts [46], individuals surveyed to estimate the VSL and VSLY may consider personal income impacts when answering the survey questions; therefore, when using the VSL/VSLY, the inclusion of productivity impacts on individuals may involve issues of double-counting.

*Framework recommendation* Health benefits stemming from prevention interventions should be quantified using an appropriate measure of health-related quality of life (DALY or QALY). This enables the translation of preventive health interventions from a CUA framework to a CBA framework. The health benefit can then be valued using the VSLY using a value of A$ 303,531 in the primary analysis and a high value of A$ 315,732 and low value of A$ 88,136 (in 2017 values). For sensitivity analyses using the health sector perspective, a wide range of QALY/DALY values should be used. The DALY and QALY quantify both morbidity and mortality impacts of interventions; however, given that the VSLY is estimated from the VSL, it may only be relevant to the mortality component of health interventions. Therefore, additional sensitivity analyses should be performed using the VSLY to value the life-years gained by the preventive health policy. The difference between the primary analysis and this sensitivity analysis will demonstrate whether the morbidity component of the QALY/DALY gain is an important consideration for an intervention. This may highlight contradictory results when conducting a CUA and CBA for the same intervention.

Preventive health interventions may have important spillover effects into other sectors [16]. Logic models should be developed to identify all potential impacts (economic, social and environmental) of the preventive health intervention. Other government CBA guidelines and completed analyses could identify appropriate methods to quantify and value impacts across sectors (for example, an evaluation of an active transport intervention’s impact on reduced car dependence can use Transport for NSW parameter values [70] to estimate the environmental benefits related to each kilometre reduction in car use). Although all impacts should be identified, steps to quantify and value these impacts should be based on logic models to ensure that the evaluation effort is commensurate with the likely size of the impact.

To maintain consistency with other health interventions, and because other relevant government guidelines do not routinely quantify productivity impacts, the Framework recommends productivity impacts are excluded in the primary analysis. In sensitivity analyses, to avoid double-counting with the valuation of VSLY, the direct productivity impacts (on individuals) should be excluded, but the indirect impact on employers should be included. It is recommended that the friction cost approach (FCA), which estimates short-term productivity losses incurred by employers in replacing a lost worker [80], be used to estimate the productivity impacts. Using highly individualized data to estimate productivity impacts may have adverse equity impacts [46], and therefore average gender-free wage rates should be used. Optional additional analyses using more individualized data could be undertaken; however, a full discussion of the distributional impact of the analyses should be included.

### What decision rules should be included in the Preventive Health CBA Framework?

All reviewed CBA guidance documents [4, 7, 8, 33, 37, 45, 48] report that the social net benefit of a programme should be demonstrated using measures of either net present value (NPV), benefit–cost ratio (BCR) or both. However, there is disagreement on the outcome of choice when the ranking of interventions differs between the two metrics. Federal government guidance [33, 45] either recommends only using the NPV or advises the cautious use of BCR. This contrasts with several of the NSW government guidelines [4, 8, 37] that recommend that the BCR should be the basis of decision-making when differences in ranking emerge between the NPV and BCR.

*Framework recommendation* To maintain consistency between government agencies, both the NPV and the BCR should be presented. However, analysts should take care in the accounting of benefits resulting from the intervention. The decision to account for avoided costs on the cost or benefit side of the equation in a CBA will not impact the NPV results, but the BCR will differ. In CEA and CUA of health interventions, avoided costs resulting from the intervention are accounted for on the cost side of the equation. The Framework recommends using the conventions reported in several government guidance documents [4, 8, 37, 81], where all impacts (including resource and health consequences) resulting from a project are counted as benefits, regardless of whether they are positive or negative [8], to ensure that BCRs are calculated consistently.

### How should uncertainty be incorporated into the Preventive Health CBA Framework?

All guidance documents emphasize the importance of testing the impact of key assumptions and variables in sensitivity analyses. Various governments’ guidance documents [4, 8, 33, 49] suggest that one-way sensitivity analyses, scenario analyses (including best/worst-case analysis) and probabilistic sensitivity analyses (using Monte Carlo simulations) are appropriate methods to quantify the variability in the results. NSW Health Infrastructure [37] and Infrastructure Australia [48] focus recommendations on identifying realistic upside and downside scenarios for analysis.

CBA guidance documents often make a distinction between risk and uncertainty [4, 37, 45, 48, 82]. “Risk” is often described as a parameter with known variability, and therefore the probability of alternative outcomes can be estimated, whereas “uncertainty” describes more vague assumptions and unknown outcomes in the future. The Handbook of CBA [45] notes that in practice, the distinction is subtle. Often in health intervention modelling, the range of possible values for variables that are accurately measurable (risk of disease, response rates, etc.) and more vague assumptions (e.g. based on expert opinion) are all incorporated in multi-way uncertainty analyses to predict the range of possible outcomes [31]. This means that although current practice for CBA and health-related CUA/CEA is similar, using the term “uncertainty analysis” may indicate less accurate parameter estimates compared to the term “risk assessment”. PM&C [82] makes an additional distinction that “risk” and “uncertainty” refer to hazardous events. However, in the practice of economic appraisal of health interventions, the term “uncertainty” refers to any reduction in confidence in a conclusion and applies equally to favourable and unfavourable events.

*Framework recommendation* All input parameters, plausible distributions and sources should be clearly documented. More extensive sensitivity analyses should be undertaken for variables that are likely to have large impacts on the results and those based on less reliable data. It is recommended that the term “sensitivity analysis” be used rather than “uncertainty analysis”. Probabilistic sensitivity analyses (using Monte-Carlo simulations and bootstrapping techniques) should be undertaken to assess the range of likely BCR results given the variability in the input parameters. Given that there are many phrases in epidemiology that use the term “risk” (e.g., risk factors, relative risk, risk ratios), it is recommended that the phrase “risk analysis” not be used in health-related CBA.

### How should equity, distributional impacts and other considerations be incorporated into the Preventive Health CBA Framework?

All CBA guidelines acknowledge that the equity objective of government intervention is not the primary goal of CBA and should not be incorporated into the technical NPV or BCR results. However, all CBA guidelines report that the distributional impact of the proposed policies should be considered in the decision-making process. PM&C [33], NSW Treasury [4] and Transport for NSW [8] recommend the use of distributional analysis to supplement the CBA results in order to demonstrate to decision-makers the winners and losers of a programme; however, the application of distributional weights is not recommended. NSW Health Infrastructure [37] recommends that the distribution of costs and benefits across stakeholders be reported, but does not recommend any specific methods. The PBAC [31] does not suggest any methods to demonstrate the access or equity impacts of interventions; however, it is reported that equity across factors such as age, socioeconomic status and geography are considered in PBAC decision-making. The NSW Health Centre for Epidemiology and Evidence [49] advises that decision-makers need to assess the results of the analysis alongside data on equity and mentions several methods for assessing equity impacts, but reports that these methods are rarely used in practice.

*Framework recommendation* It is important to consider the equity and distributional impacts of preventive health policies in resource allocation decision-making. However, in order to ensure that the CBA results remain transparent [46], the equity impacts should not be incorporated into the results in the primary analysis. The equity and distributional impacts should be fully described, and disaggregated impacts across relevant subgroups (by socioeconomic status, cultural background and geographical location) should be quantified where appropriate.

There are several factors related to preventive health interventions that may be important for decision-makers to consider which are not captured in technical CBA results. Examples include the acceptability of the intervention to various stakeholders, feasibility of implementation and strength of evidence of intervention effectiveness [57]. These should be identified and reported either qualitatively or quantitatively. Unintended impacts of interventions are important to consider and should be identified in the intervention logic models and quantified where appropriate.

### Reporting requirements for the Preventive Health CBA Framework

All reviewed guidance documents recommend that all critical assumptions and inputs are reported with supporting evidence where available [4, 7, 8, 31, 33, 37, 45, 48, 49]. All relevant cost and benefit categories, sensitivity analyses and distributional impacts for all the options assessed should be documented. PM&C [33] recommends presenting costs and benefits in three categories: monetized, quantified but not monetized, and qualitative but not quantified or monetized. The PBAC [31] provides highly detailed reporting guidelines and stipulates that the methods used to identify input data should be robust, transparent and clearly justified. A minimum data set for inputs and disaggregated results is identified. The NSW Health Centre for Epidemiology and Evidence [49] recommends reporting based on the Consolidated Health Economic Evaluation Reporting Standards [83].

*Framework recommendation* The options for appraisal, assumptions and inputs used in the analysis with reference to evidence should be accurately and transparently documented. Results should be disaggregated by sector to enable the translation of results from a CBA to a CUA/CEA framework. Impacts should be categorized by method of measurement as recommended by the PM&C [33]. Full documentation of sensitivity analyses and distributional impacts should be included, with accurate interpretation of findings.

### Differences in typology between guidelines

In addition to the terminology already mentioned in the Framework, there are differences in the definitions of other terms and how they are used in the different guidelines. NSW Treasury [4] defines “direct costs” as costs that directly impact producers and consumers of goods and services associated with the project, and “indirect costs” as costs impacting third parties not directly involved in the consumption or production of the goods/services. However, this differs from the NSW Health Centre for Epidemiology and Evidence [49] definition, which reports “direct costs” as costs incurred in running the health programme (staff time, drugs, materials, etc.) and “indirect costs” as the economic burden incurred by individuals, families and the community associated with illness.

NSW Treasury [4] uses the term “economic appraisal” for ex-ante analyses and “economic evaluation” for ex-post analyses. In the PBAC [31] guidelines, the term “economic evaluation” is used for all submissions to PBAC; the majority of these are ex-ante analyses. In the practice of economic appraisal of health interventions, economic evaluation is defined as requiring two components: (1) the analysis needs to be a comparative assessment of alternative courses of action, and (2) both the costs and consequences of the choices need to be analysed [84], and used to describe both ex-ante and ex-post analyses.

*Framework recommendation* Until there is harmonization of terminology for all economic analyses, the definitions used by state treasury departments should be followed. When communicating the results to a health sector audience, the various meanings of these terms should be defined.

## Discussion

Australian governments are committed to using economic evidence in the form of CBA to aid decision-making [1–6, 40, 41]. Given that preventive health interventions are often cross-sectoral and require Cabinet approval, a CBA framework may be required for decision-making. The Preventive Health CBA Framework is a set of recommendations for the economic appraisal of preventive health interventions to aid government decision-making. The need for these CBA guidelines is a result of the challenges associated with accurately assessing the credentials of preventive health interventions using conventional CUA/CEA frameworks or current CBA guidelines. Frameworks and guidelines can enhance the replicability, comparability, credibility and usability of CBA results [25]; however, it is recommended that practice guidelines are not viewed as requirements that the analyst must apply indiscriminately.

The Framework balances the need for consistency with central guidelines, while incorporating the unique features of prevention interventions by defining a reference case for Cabinet submissions and additional sensitivity analyses. This allows the analyst to demonstrate the impact of key parameters, such as the time horizon or the discount rate, on the economic credentials of prevention interventions. To inform resource allocation decision-making within the health sector, it is important that preventive health policy analyses are comparable with other health interventions assessed using a CUA or CEA framework. This will allow the assessment of the value for money of interventions across the spectrum of health interventions from primary prevention and secondary prevention through to treatment. To facilitate this, the Framework recommends that health benefits are estimated in QALYs and DALYs prior to being monetized and that sensitivity analyses be included that (1) use a health sector perspective; (2) use various time horizons, discount rates and VSLY values; and (3) disaggregate impacts across sectors.

The need to comply with varying evaluation conventions is not a unique feature of health interventions, with analyses across different contexts being associated with different CBA communities of practice [25]. It is important that the audience for the analysis is carefully considered, as the use of multiple frameworks may be a source of confusion for politicians and bureaucrats alike [10], and may diminish their impact.

Using the NSW government as an example, the Framework reference case recommendations are largely consistent with the NSW Treasury CBA guidelines [4] to promote consistency in Cabinet decision-making. However, there are several inconsistencies and issues that need to be resolved to improve the comparability of CBA across various sectors. Firstly, parameters such as the VSL/VSLY that are key drivers of CBA results [86–88] should be the focus of parameter harmonization efforts. The Framework recommends using results from a recent systematic review [76]; however, this needs to be deliberated by the departments that currently use varying values. Developing a parameter database that is regularly updated may be an effective way to ensure consistency of parameter values; it may also assist analysts in incorporating intersectoral impacts into CBA. However, the cost of developing and maintaining a database is a limitation [10]. Secondly, discrepancies in typology can cause confusion, particularly when communicating results to varied audiences. Therefore, as experience with conducting CBA for preventive health interventions increases, there needs to be further discussion with Treasury and other government line agencies around the appropriate typology and agreement on the meaning of terms such as “base case” versus “comparator”, “appraisal” versus “evaluation”, and “risk” versus “uncertainty”. The definitions of terms such as “direct” and “indirect” impacts also need clarification.

The key aim of economic analyses is to inform the efficient allocation of resources; however, the development of consistent CBA frameworks is not the only requirement for increased efficiency in government decision-making. Firstly, in addition to applying the CBA framework, the analyst needs to conduct high-quality analyses using sound applied economic evaluation concepts, theories and practices [44–46, 84]. Secondly, politicians engaged in the Cabinet process and bureaucrats supporting them need to understand CBA and value its contribution to the assessment of allocative efficiency. According to Dobes et al. 2016 [8], a cultural change in government where bureaucrats volitionally use and value CBA is needed and is likely to be more effective than mandated guidelines. This will require increased resources and expertise in the conduct of CBA within government departments [11].

CBA is a tool used to inform decisions related to allocative efficiency, given a particular distribution of income in a society. CBA does not provide any judgement on whether the current distribution of income is equitable, and in fact, is biased in favour of the existing distribution [45]. From a welfare economics tradition, CBA guidelines [3, 32] specify that outcomes should be valued based on the preferences of the individuals or the firms that experience the outcomes. However, this may bias outcomes against those who have a lower ability to pay [45]. From an extra-welfarist perspective, where the focus is on health outcomes, the PBAC [12] recommends that the scoring algorithm used to value health states (to calculate QALYs) be based on representative Australian preference weights. This implies that the preferences of interest are those of the general Australian public, not individuals who experience the outcome. Further dialogue amongst the public and decision-makers related to whose values should count (individuals affected by intervention, the general population, third-party experts or decisions-makers) for government resource allocation decision-making is required. All CBA guidelines reviewed recommended that the distribution of impacts be presented alongside the technical CBA results [4, 7, 8, 33, 37, 45, 48, 49]. Further research is required to better understand how these impacts are considered in decision-making and the methods to better assist decision-makers in using distributional analyses that accompany CBA [89].

The discount rate used in economic analyses is an important topic that remains hotly debated nationally and internationally, with little consensus amongst academics and government departments [29, 59, 90]. Key topics of debate include whether the basis of the discount rate for public policy appraisals should be the opportunity cost of capital, the social rate of time preference, or variations/combinations of the two [4, 33, 59, 62, 63, 91]. There is also little consensus on how to calculate the actual discount rate to be used and whether the rate should vary over time, by sector or by level of project risk, and whether it should incorporate equity considerations for future generations [29, 59, 62, 91]. Despite the lack of consensus, the choice of discount rate remains particularly important for preventive health interventions, where the benefits may only be realized many years into the future [19]. Given the lack of theoretical and methodological clarity, the Framework recommendation is based on the argument that the current discount rates endorsed by Australian governments are too high and are based on historical values, and therefore a lower rate of 3% used in preventive health intervention evaluations is recommended. The basis for variations in the discount rate across Australian jurisdictions is unfounded and therefore clearly an area for further research and consensus-building across all governments in Australia. The federal government should take leadership on this and investigate and update the recommended discount rate on a regular basis [66].

The Preventive Health CBA Framework was developed using the NSW government as an example; however, the findings from this study have broader application. The Framework considers key factors such as discount rates, time horizons and intersectoral impacts that are crucial to consider when conducting economic appraisal of preventive health interventions—these factors are universal, irrespective of jurisdiction. Adjustment of the Framework to suit the needs of other Australian jurisdictions and international contexts would require a review of central agency recommendations to ensure consistency across key parameters. Australian and international applications of the Framework by researchers and governments are an important next step to assess the usefulness of the Framework and to inform its ongoing development and refinement for context. An international community of practice for the application of CBA for preventive health interventions will result in improved analyst skills, methodologies and acceptability amongst decision-makers.

Limitations of the methods used to develop the CBA Framework included the reliance on the relevancy ranking of the government website search functions. It is also possible that relevant documents were missed at the initial title and short-text screening that was conducted by one author. Another limitation is that only documents that were publicly available online were included, and therefore documents in development were missed in the review. Further, a systematic assessment of the application of current government economic evaluation guidelines was out of scope for this study and should be the focus of future research.

## Conclusions

The Preventive Health CBA Framework is based on a review of the most relevant and contemporary Australian government CBA and other health economic evaluation guidance documents, and the published literature. As with all frameworks and guidelines, the Framework will need to be tested extensively, periodically reviewed and refined [12]. The instantaneous change of evaluation practice to use a CBA framework for preventive health interventions is unrealistic, and there are several hurdles. Firstly, Australian governments need to accept a CBA framework for the assessment of preventive health interventions as complementary to traditional CUA/CEA methods for health-related economic appraisals. Secondly, there needs to be commitment from health departments to embed the use of economic evidence in decision-making and to invest in capacity-building so that the Framework can be trialled across various policies. This will allow the assessment of the specifications for the primary analysis and sensitivity analyses in terms of their ability to accurately assess the credentials of preventive health interventions. The use of the Framework will facilitate the development of empirical evidence that can be used in future evaluations. Thirdly, the acceptance and use of the Framework will also require treasury departments to better understand the unique features of preventive health interventions and to accept the deviations away from the current treasury guidance. This process is likely to be time- and resource-intensive for both health and treasury departments and will require a close and effective working relationship between them. Despite the large commitment required, governments are gradually moving towards mandated CBA processes [4, 33, 40, 41], and therefore the health sector must adapt so that preventive health analyses are useful to both central and sector-specific decision-making.

## Supplementary Information

**Additional file 1:** Definition of terms. Provides a definition of key terms used in the article.

**Additional file 2:** Document search flow chart.

**Additional file 3:** Data relevant to cost–benefit analyses extracted from government documents. Provides data relevant to cost–benefit analyses that were extracted from the 30 government documents that were reviewed but were not the main guidance document used to formulate the recommendations.

## Data Availability

All data generated or analysed during this study are included in this published article and its Additional information files.

## Abbreviations

BCR: Benefit–cost ratio
CBA: Cost–benefit analysis
CEA: Cost-effectiveness analysis
COAG: Council of Australian Governments
CUA: Cost-utility analysis
DALYs: Disability-adjusted life-years
FCA: Friction cost approach
ICER: Incremental cost-effectiveness ratio
LGA: Local government area
MCA: Multi-criteria analysis
NPV: Net present value
NSW: New South Wales
OBPR: Office of Best Practice Regulation
PBAC: Pharmaceutical Benefits Advisory Committee
PBS: Pharmaceutical Benefits Scheme
PM&C: Prime Minister and Cabinet
QALYs: Quality-adjusted life-years
RP: Revealed preference
SP: Stated preference
VSL: Value of a statistical life
VSLY: Value of a statistical life-year
WACC: Weighted average cost of capital
WEB: Wider economic benefits
