# Supplementary material for: A cost–benefit analysis framework for preventive health interventions to aid decision-making in Australian governments
Source: Health Res Policy Syst. 2021 Dec 19;19:147. doi: 10.1186/s12961-021-00796-w (PMC8684630; doi:10.1186/s12961-021-00796-w)
Supplement: Supplementary file 1 — Additional file 1: Definition of terms. Provides a definition of key terms used in the article. [file 12961_2021_796_MOESM1_ESM.docx]

**Additional file 1: Definition of terms**

| Absenteeism | Reduced productivity resulting from a worker being absent from work. |
| --- | --- |
| Benefit cost ratio | The relative costs and benefits of a proposed project (benefits divided by the costs). If the ratio is greater than 1, then the benefits outweigh the costs and therefore the project should be funded. |
| Cost-benefit analysis | Economic evaluation technique used to assess the costs and benefits of interventions, where both the benefits and the costs are monetised. |
| Cost-effectiveness analysis | Economic evaluation technique used to assess the costs and benefits of interventions, where the benefits are estimated in natural units, e.g. cost per life year saved. |
| Cost-utility analysis | Economic evaluation technique used to assess the costs and benefits of interventions, where the benefits are estimated using a generic measure of health that incorporates the quality and quantity of life gained, e.g. cost per quality adjusted life year gained. |
| Decision-Making Approach | A normative theory (less developed than Welfarism and Extra-welfarism) that asserts that the social decision-maker should determine society’s objectives and therefore the cost-benefit analysis should capture the range of impacts relevant to the decision-maker. |
| Disability adjusted life years | A generic measure of health burden that incorporates the years of life lost due to premature mortality and years lost due to disability. |
| Extra Welfarism | Normative economic theory used in the health sector, with the view that health sector interventions should aim to maximise health rather than individual utility. |
| Friction cost approach | An approach to calculating productivity loss. The method reflects the cost to employers by valuing the period required to replace the sick worker who is no longer able to work (the friction period). |
| Economic multipliers (e.g. income multipliers) | The flow on impacts on income or employment resulting from a project (usually infrastructure projects). These impacts should not be included in cost-benefit analysis because they are an indication of economic activity rather than a measure of societal benefits. |
| Joint risk factor adjustment | Adjustments made to account for the multiple risk factors that contribute to a disease outcome. |
| Market risk premium | The quantitative measure of the extra return expected in the market compared to the risk free rate. The market risk premium and the risk-free rate of return can vary over time and are used to calculate the opportunity cost of capital. |
| Multi-criteria analysis | A technique that explicitly outlines the criteria used in decision-making and the relative importance attached to each of them. Different intervention options can then be scored against each of the criteria to identify the most promising options. |
| Probabilistic sensitivity analyses (Monte-carlo simulations) | Investigates the sensitivity of the evaluation results due to parameter uncertainty. Multiple input parameters are varied simultaneously based on their defined distributions. Random sampling of the defined distribution is used to set the value of the input parameters. The model is then run and the results are recorded. This is undertaken multiple times (1000 times or more) to produce a distribution of results. Monte-Carlo simulation is one of the methods of undertaking probabilistic sensitivity analyse. |
| Net present value | The present value of future costs and benefits calculated using a specific discount rate. The net present value of the costs are subtracted from the benefits. If the net present value is positive, then the benefits of the project outweigh the costs and the project should be funded. |
| One-way sensitivity analysis | Investigates the sensitivity of the evaluation results to one input parameter. The value of a single parameter is varied within a pre-defined range and the impact on the results is recorded. Also called univariate sensitivity analysis. |
| Opportunity cost of capital | The rationale for the social discount rate that considers the opportunity cost associated with government investment that displaces other investments in the market. The market risk premium and the risk-free rate of return can vary and are used to calculate the opportunity cost of capital. |
| Orthodox Welfarism | Normative economic theory that views social welfare as a function of individual preferences and utilities. Under this theory, the goal of social policy is to maximise individual utility. |
| Potential impact fraction | The proportional reduction in disease risk when the distribution of a risk factor for that disease changes. |
| Presenteeism | Reduced productivity resulting from a worker present at work but not fully functioning often due to poor health. |
| Quality adjusted life year | A generic measure of health that incorporates the quality and quantity of life gained by an intervention. |
| Revealed preference | Valuation of goods and services based on observations of actual purchasing decisions. |
| Risk free rate of return | The interest rate paid on investments with zero risks. In practice, long term government bond yields have been used to estimate this. The market risk premium and the risk-free rate of return can vary and are used to calculate the opportunity cost of capital. |
| Scenario analysis | The simultaneous change in multiple parameters to assess the impact on the evaluation results. The changes to the parameters may correspond to best case or worst case scenarios. Also called multi-way sensitivity analyses. |
| Social discount rate | The discount rate that is applied to compare projects with costs and benefits that occur in different time periods. Discounting is applied because individuals have a time preference to consume sooner rather than. Another rationale for discounting is that government investment displaces investment that would have occurred somewhere else in the economy. These two rationales give rise to the two approaches to defining the discount rate being the Social Rate of Time Preference and the Opportunity Cost of Capital. |
| Social Rate of Time Preference | The rate at which society is willing to trade current for future consumption. The rate of return of long term government bonds have been used to estimate the Social Rate of Time Preference. |
| Stated preference | Valuation of goods and services based on surveys of people’s stated preferences for hypothetical scenarios. |
| Time horizon | The duration over which the costs and benefits of an intervention are captured. The time horizon should be long enough to capture all important costs and benefits of an intervention. However longer time horizons are likely to be reliant on less robust data and therefore require more assumptions. |
| Value of a statistical life | An estimate of individual’s willingness to trade wealth for small fatality risk reductions. It is a key parameter for the evaluation of public policies related to health and safety. |
| Value of a statistical life year | The value of one additional year of life expectancy. This can be calculated using the value of a statistical life or using stated preference studies. |
| Wider economic benefits | Improvements in economic welfare that are not easily captured in cost-benefit analyses. These benefits are often second round impacts that should not be included in CBA and care should be taken not to double count these benefits with those already captured in the CBA. The largest source of wider economic benefits relate to infrastructure projects that result in ‘agglomeration economies’ where productivity benefits are realised by firms being in close proximity to each other. |
| Weighted average cost of capital | The cost of all sources of capital calculated by the weighted average debt and equity costs. It can be used as a rate by industry to determine whether or not make a specific investment based on its predicted rate of return. |
