# Supplementary material for: A cost–benefit analysis framework for preventive health interventions to aid decision-making in Australian governments
Source: Health Res Policy Syst. 2021 Dec 19;19:147. doi: 10.1186/s12961-021-00796-w (PMC8684630; doi:10.1186/s12961-021-00796-w)
Supplement: Supplementary file 2 — Additional file 2: Document search flow chart. [file 12961_2021_796_MOESM2_ESM.docx]

**Additional file 2: Document search flow chart**

Records identified:

Federal government website (n = 2566)

NSW government central agencies websites (n = 792)

NSW government line agencies websites (n = 1876)

Recommended by NSW informants (n = 6)

**Identification**

Records excluded (n = 5169)

Records with titles screened

(n = 5240)

Reports excluded due to not reporting methods or values for use in cost-benefit analysis (n = 18)

Document superseded by a more recent document (n = 14)

**Screening**

Reports assessed for eligibility

(n = 71)

**Documents included (n = 39)**

Cost-benefit analysis guidance documents (n = 9)

Other documents providing guidance on cost-benefit analysis methods and values (n = 30)

**Included**
