# Supplementary material for: A cost–benefit analysis framework for preventive health interventions to aid decision-making in Australian governments
Source: Health Res Policy Syst. 2021 Dec 19;19:147. doi: 10.1186/s12961-021-00796-w (PMC8684630; doi:10.1186/s12961-021-00796-w)
Supplement: Supplementary file 3 — Additional file 3: Data relevant to cost–benefit analyses extracted from government documents. Provides data relevant to cost–benefit analyses that were extracted from the 30 government documents that were reviewed but were not the main guidance document used to formulate the recommendations. [file 12961_2021_796_MOESM3_ESM.docx]

**Additional file 3: Government documents providing data on cost-benefit analysis methods and values**

| **Jurisdiction and Department** | **Title** | **Aims of document** | **Guidance related to CBA** |
| --- | --- | --- | --- |
| Federal  PM&C (OBPR) [1] | Australian Government Guide to Regulatory Impact Analysis (2020) | High level document outlining the process for undertaking regulatory impact statements which are mandatory for Cabinet submissions. | CBA is a key component of a RIS however specifications are not reported.  Provides some information on the appropriate comparator, the costs for inclusion, the decision rules and the reporting of distributional impacts. |
| Federal  PM&C (OBPR) [2] | User guide to The Australian Government guide to Regulatory Impact Analysis (2020) | Outlines the principles for policy making related to regulation and the process for developing a RIS. | None. |
| Federal  PM&C (OBPR) [3] | Best Practice Consultation. Guidance note (2020) | Best practice guidance on stakeholder consultation which is a key component of a RIS. | Consultation should be used to identify the policy alternatives available. |
| Federal  PM&C (OBPR) [4] | Council of Australian Governments’ Regulation Impact Statement Preliminary Assessment: Is a RIS required? | High level guidance to assess whether a RIS is required for COAG decision-making. | Advice on the range of compliance costs that should be considered when completing a RIS. |
| Federal  PM&C (OBPR) [5] | Community organisations (2020) | Guidance on the key factors to consider when a RIS impacts community organisations. | None. |
| Federal  PM&C (OBPR) [6] | Competition and Regulation (2020) | Provides guidance on the additional 'tests' to be outlined in an RIS when the preferred regulatory option restricts competition. | It is unclear what the decision rule is if there is a feasible alternative policy option that does not restrict competition but results in lower net benefits than the option that does restrict competition but has the greatest net benefits.  Given that the impacts of reduced competition are difficult to quantify, these can be described in qualitative terms e.g. potential for longer wait times, higher prices etc.  The significance of the costs should be assessed in reference to the specific market rather than the whole economy. |
| Federal  PM&C (OBPR) [7] | Distributional Analysis (2020) | To provide guidance on how to perform distributional analysis as part of the RIS. | Distributional analysis is a second step in the analysis and should not be included in the net benefits calculation.  Distributional analysis should be performed when there is a significant impact on different groups or when the aim of the policy has a redistributive objective.  The following cohorts are the examples provided however the list is not exclusive: age, gender, disability, Indigeneity, regional/geography, income/wealth level, business size, cultural background/immigration status. |
| Federal  PM&C (OBPR) [8] | Environmental valuation (2020) | Provides high level guidance on incorporating environmental impacts and uncertainty into RIS. | The impact of the environmental asset on outcomes that can be directly valued by individuals should be described and monetised where possible.  A declining long-term discount rate can be used for time horizons over 30 years: 31-75 years - 5.4%; 76-125 years - 4.8%; 126-200 years - 4.3%; 201-300 years - 4.0%; 301+ years - 3.7% |
| Federal  PM&C (OBPR) [9] | Independent Reviews, RIS-like processes and the Regulation Impact Statement requirements (2020) | Provides guidance on the special case of proposals that have been assessed in an Independent Review or other RIS like process and therefore a RIS is not required. | None. |
| Federal  PM&C (OBPR) [10] | Individuals (2020) | Provides guidance on how to quantify regulatory costs on individuals in a RIS. | Provides a value of $32 per hour for an individual's time when not in employment (leisure time). |
| Federal  PM&C (OBPR) [11] | Overview of the changes to the Australian Government  Regulatory Impact Analysis framework (2020) | Outlines the changes to the RIS from the previous version. | None. |
| Federal  PM&C (OBPR) [12] | Post-implementation Reviews (2020) | Provides guidance on when and how to prepare a post-implementation review. | The objective of the post implementation review is to assess the appropriateness, effectiveness and efficiency of implemented regulations and the impacts on all stakeholders should be discussed. If a full quantitative CBA is not possible, the material impacts on the key stakeholders should be measured. Distribution of costs and benefits including jurisdictional differences should be noted. |
| Federal  PM&C (OBPR) [13] | Australian Government Regulation Impact Statement Preliminary Assessment Form: Is a RIS required? (2020) | Provides guidance on the Preliminary Assessment form which is used by OBPR to determine whether a RIS is required (i.e. determining whether the policy (not involving Cabinet) is minor or machinery in nature). | None. |
| Federal  PM&C (OBPR) [14] | Regulatory Burden Measurement Framework (2020) | Provides guidance on calculating the regulatory cost to be included in a RIS. | Details of the costs that should be included and excluded as regulatory costs in an RIS are provided. These costs should be disaggregated by sector and whether the costs accrue to business, community organisations or individuals.  Reports that costs should be presented as average annual costs in real terms over a default period of 10 years.  Provides a general hourly labour cost of $73.05 which includes average wage adjusted to include income tax and scaled up by a multiplier of 1.75 to include on-costs. This default value should be used for regulations that impact various sectors, or when sector specific labour rate calculation will add undue complexity to the costing process. Leisure time for individuals is valued at $32 per hour. |
| Federal  PM&C (OBPR) [15] | Regulation Impact Statement Policy Options (2020) | Provides guidance on selecting policy options for RIS. | When choosing options for appraisal, all practical policy alternatives should be considered. The RIS needs to include at least three options, with one option being a non-regulatory option. It is not clear whether "status quo" is considered an option or the comparator. There are also several circumstances when just one option can be considered against the status quo: when the policy is related to an election commitment and when it is related to international agreements. |
| Federal  PM&C (OBPR) [16] | Small Business (2020) | Provides guidance on the key factors that impact small businesses that should be considered in RIS. | None. |
| Federal  PM&C (OBPR) [17] | Sunsetting Legislative Instruments (2020) | Sets out the requirements for RIS for Sunsetting regulatory instruments. | None. |
| Federal  PM&C (OBPR) [18] | Trade Impact Assessments (2020) | Sets out the requirements for when a trade impact statement is required to be incorporated into the RIS. | None. |
| Federal  PM&C (OBPR) [19] | Risk Analysis in Regulation Impact Statements (2020) | Provides guidance on how to evaluate regulations aimed at managing risk in RIS. | The term risk is used to characterise hazardous events. A distributional analysis of risks is recommended.  Undesirable outcomes of regulation should be incorporated into sensitivity analyses quantitatively using 'best case' and 'worst case' scenarios and probabilistic modelling. When these can't be quantified, they should be described qualitatively. |
| Federal  PM&C (OBPR) [20] | Best Practice Regulation Guidance Note. Value of statistical life (2019) | Provides guidance on how the value of a statistical life (VSL) measure should be used in CBA. | Reports that willingness to pay is the appropriate method to estimate the value society places on reducing the risk of dying for a statistical life of a young adult with at least 40 years of life remaining.  The value it based on Australian and international research and international guidelines on VSL and expressed in 2007 values. Although outdated, it is reported to be the best estimate of VSL and value of a statistical life year (VLY). The 2007 values are inflated using the ABS Wage Price Index data to 2019 values (VSL of $4.9 million and a VSLY of $213,000).  The VSLY is calculated using a private time preference discount rate of 3%, whereas the discount rate for the CBA is 7%.  Reports that the VSL should vary according to the characteristics of people affects and the type of risk. However the recommended value is based on a review of the literature with only one value provided citing that it would be too costly to estimate this value for each proposal.  Sensitivity analyses should focus on the parameters related to estimating the number if lives or life years and cost of the intervention should be varied rather than using arbitrary values in sensitivity analyses. |
| Federal  Department of Finance and Deregulation (OBPR) [21] | Influencing Consumer Behaviour: Improving Regulatory Design (2012) | Outlines behavioural economics as a tool to be considered when designing regulatory interventions aimed to change consumer behaviour. | None. |
| Federal  Productivity Commission [22] | Valuing the Future: the social discount rate in cost-benefit analysis (2010) | Investigates the appropriate discount rate for government CBA. The views expressed in this paper is of the visiting researcher and not that of the Productivity Commission. | Reports that the theoretically sound discount rate should consider the source of project funds and therefore should be the weighted average of the consumption rate, investment rate and marginal cost of foreign funds and adjusted for irreducible risk. But in practice there is limited data to inform this calculation. In practice, the appropriate rate is the social opportunity cost of capital based on the marginal rate of return on capital (which includes a market risk premium) adjusted to reflect tax distortions and foreign borrowing. A central value of 8% with sensitivity analysis using 3% and 10% is recommended. 3% represents the weighted average riskless rate of return, 8% the weighted average rate of return, and 10% the rate of return for a riskier project that reflects the marginal productivity of capital during 2000. When the discount rate impacts the sign of the NPV or the ranking of options, then more consideration should be given to the appropriate discount rate for the specific project.  Defines benefits as all the desirable effects of a policy and costs as all the undesirable effects. This is not consistent with other costing conventions and therefore could result in inconsistent BCR. |
| NSW  Health Infrastructure [23, 24] | Toolkit for cost-benefit analysis of health capital projects Volume 1 and 2 (2017) | Provides explanatory notes on the identification and calculation of the benefits of health infrastructure capital projects. | Reports being consistent with the NSW Treasury CBA guidelines.  Although the assumptions associated with the calculations are made explicit - there is little evidence to support many of the assumptions used.  Reports that wider economic impacts such as agglomeration should be included as indirect economic impacts. However both the NSW Health Infrastructure guidance and the NSW Treasury guidance report that these should not be included in the primary CBA.  VSL/VSLY and travel cost input values reported.  Provides a summary of the social, economic and environmental impacts that should be described qualitatively. |
| NSW  Health - Centre for Epidemiology and Evidence [25] | Commissioning Evaluation Services: A Guide (2019) | Supports NSW Health in the commissioning of population health program evaluations. | None. |
| NSW  Transport for NSW [26] | Transport for NSW. Economic Parameter Values (2020) | Provides recommended parameter values for the common costs and benefits used in transport CBA. | Provides various input values for transport CBA including VSL and the valuation of health benefits associated with active transport. |
| NSW  Transport for NSW [27] | Transport for NSW. Business Case Guide (2020) | Provides guidance on the steps and content required for major transport investments business cases. | Outlines where in the business case high level and detailed CBA are required. A strategic business case and a final business case are defined.  The short list of options require a detailed CBA in the final business case. Key features of the long list of options need to be outlined in the business case and high level CBA undertaken. The purpose of the CBA at this stage is to systematically analyse the long list of options to develop the short list. If it is not practical to provide CBA for long list of options, the reasons for this must be provided. For the short list of options included in the final business case, the full CBA results are required along with benchmarking of results against other similar past projects. |
| NSW  Treasury [28] | NSW Government Guide to Better Regulation (2019) | Outlines the concepts and processes for good regulatory practice within NSW Government agencies. | CBA is a component of the Better Regulation Statement. It is required for all significant regulatory proposals (e.g. major new regulatory initiative, significant impact on NSW community, significant compliance costs, impose on competition, or significant administrative cost to government).  The costs and benefits must be monetised as much as possible. The options considered should include the 'status quo' and non-regulatory options. The assessment of costs and benefits should reflect the significance of the proposal, and detailed costs and benefits are not required for options that are clearly less optimal.  The preferred option meets the policy objectives and has the greatest net benefit or least cost to the community. |
| NSW  Treasury [29] | NSW Gateway Policy (2017) | Outlines the principles and processes of the Gateway policy (a project assurance process). It asses programs based on risk and conducts independent reviews at key decision points in the project lifecycle to identify risks and provides advice to strengthen project justification, delivery and benefits realisation. | Although no guidance is provided on CBA, it is reported that the Gateway process is designed to support the delivery of value for money related to major government investments by providing independent review of projects at major decision points.  The definition of risk for the Gateway process is related to total cost of the program, level of government priority, complexity, agency capability and the criticality of the service.  For proposals that fall under the Gateway policy, the preliminary CBA as part of a preliminary Business case, and the detailed CBA prior to Cabinet approval, will need to be reviewed as part of the Gateway review process. |
| NSW  Treasury [30] | NSW Government Business Case Guidelines (2018) | Assists government agencies develop robust business cases that are standardised, transparent, scalable and focused on outcomes in order to inform resource allocation decision-making for all types of government investment, policies and regulations. The guidelines help to develop business cases that demonstrate the proposed intervention represents value for money, is financially and commercially viable and can achieve the proposed outcomes. | The business case should include evidence from a CBA showing that the selected option maximises social welfare and delivers value for money.  Guidance for the development of the long list of options for analysis is given. The high level CBA of the long list of options is undertaken in the Strategic Business Case. The short list of options for analysis is based on the initial CBA, but also the financial appraisal. Reasons for the exclusion from the short list of options should be documented. A detailed full CBA of the short list of options is required for the Detailed Business Case. The preferred option is based on the CBA and financial appraisal. An evaluation plan (process, outcome or economic evaluation) is also required in the Detailed Business Case.  The reader is referred to the NSW Treasury CBA guidelines for details on the conduct of CBA. High level outline of CBA process is provided. |
| NSW  Infrastructure NSW [31] | Infrastructure Investor Assurance Framework: Gateway Coordination Agency Framework for Capital Projects under the NSW Gateway Policy (2020) | Provides Gateway guidelines for NSW infrastructure projects taking a risk based assurance framework. | Business case is optional for tier 3 and not required for tier 4 projects which are categorised based on level of spend and risk of the project. |
| Notes: CBA: Cost-Benefit analysis; COAG: Council of Australian Governments; OBPR: Office of Best Practice Regulation; PM&C: Prime Minister and Cabinet; RIS: Regulatory Impact Statement; VSL: value of a statistical life; VSLY: value of a statistical life year; | | | |

**References**

1. Department of Prime Minister and Cabinet. The Australian Government guide to regulation impact analysis. Second edition. In: Department of Prime Minister and Cabinet, editor. Canberra: Commonwealth of Australia; 2020.

2. Department of Prime Minister and Cabinet. User guide to The Australian Government Guide to Regulation Impact Analysis. In: Department of Prime Minister and Cabinet, editor. Canberra: Commonwealth of Australia; 2020.

3. Department of Prime Minister and Cabinet. Best Practice Consultation. Guidance note In: Department of Prime Minister and Cabinet, editor. Canberra: Commonwealth of Australia; 2020.

4. Department of Prime Minister and Cabinet. Council of Australian Governments’ Regulation Impact Statement Preliminary Assessment: Is a RIS required? In: Department of Prime Minister and Cabinet, editor. Canberra: Commonwealth of Australia; 2020.

5. Department of Prime Minister and Cabinet. Community Organisations. In: Department of Prime Minister and Cabinet, editor. Canberra: Commonwealth of Australia; 2020.

6. Department of Prime Minister and Cabinet. Competition and Regulation In: Department of Prime Minister and Cabinet, editor. Canberra: Commonwealth of Australia; 2020.

7. Department of Prime Minister and Cabinet. Distributional Analysis In: Department of Prime Minister and Cabinet, editor. Canberra: Commonwealth of Australia; 2020.

8. Department of Prime Minister and Cabinet. Environmental valuation In: Department of Prime Minister and Cabinet, editor. Canberra: Commonwealth of Australia; 2020.

9. Department of Prime Minister and Cabinet. Independent Reviews, RIS-like processes and the Regulation Impact Statement requirements In: Department of Prime Minister and Cabinet, editor. Canberra: Commonwealth of Australia; 2020.

10. Department of Prime Minister and Cabinet. Individuals. In: Department of Prime Minister and Cabinet, editor. Canberra: Commonwealth of Australia; 2020.

11. Department of Prime Minister and Cabinet. Overview of the changes to the Australian Government Regulatory Impact Analysis framework In: Department of Prime Minister and Cabinet, editor. Canberra: Commonwealth of Australia; 2020.

12. Department of Prime Minister and Cabinet. Post-implementation Reviews In: Department of Prime Minister and Cabinet, editor. Canberra: Commonwealth of Australia; 2020.

13. Department of Prime Minister and Cabinet. Australian Government Regulation Impact Statement Preliminary Assessment Form: Is a RIS required? . In: Department of Prime Minister and Cabinet, editor. Canberra: Commonwealth of Australia; 2020.

14. Department of Prime Minister and Cabinet. Regulatory Burden Measurement Framework In: Department of Prime Minister and Cabinet, editor. Canberra: Commonwealth of Australia; 2020.

15. Department of Prime Minister and Cabinet. Regulation Impact Statement Policy Options In: Department of Prime Minister and Cabinet, editor. Canberra: Commonwealth of Australia; 2020.

16. Department of Prime Minister and Cabinet. Small Business In: Department of Prime Minister and Cabinet, editor. Canberra: Commonwealth of Australia; 2020.

17. Department of Prime Minister and Cabinet. Sunsetting Legislative Instruments In: Department of Prime Minister and Cabinet, editor. Canberra: Commonwealth of Australia; 2020.

18. Department of Prime Minister and Cabinet. Trade Impact Assessments In: Department of Prime Minister and Cabinet, editor. Canberra: Commonwealth of Australia; 2020.

19. Department of Prime Minister and Cabinet. Risk Analysis in Regulation Impact Statements In: Department of Prime Minister and Cabinet, editor. Canberra: Commonwealth of Australia; 2020.

20. Department of Prime Minister and Cabinet. Best Practice Regulation Guidance Note. Value of statistical life In: Department of Prime Minister and Cabinet, editor. Canberra: Commonwealth of Australia; 2019.

21. Deregulation DoFa. Influencing Consumer Behaviour: Improving Regulatory Design In: Department of Prime Minister and Cabinet, editor. Canberra: Commonwealth of Australia; 2012.

22. Harrison M. Valuing the Future: the social discount rate in cost-benefit analysis. In: Commission P, editor. Canberra: Commonwealth of Australia; 2010.

23. Health Infrastructure. Toolkit for cost-benefit analysis of health capital projects. Volume 1. In: Health Infrastructure, editor. Sydney: NSW Government; 2017.

24. Health Infrastructure. Toolkit for cost-benefit analysis of health capital projects. Volume 2. In: Health Infrastructure, editor. Sydney: NSW Government; 2017.

25. NSW Health Centre for Epidemiology and Evidence. Commissioning Evaluation Services: A Guide In: NSW Ministry of Health, editor. Sydney, NSW: NSW Government,; 2019.

26. Transport for NSW. Transport for NSW. Economic Parameter Values. Version 2.0. In: Transport for NSW, editor. Sydney: NSW Government; 2020.

27. Transport for NSW. Transport for NSW. Business Case Guide In: Transport for NSW, editor. Sydney: NSW Government; 2020.

28. NSW Treasury. NSW Government Guide to Better Regulation In: The Treasury, editor. Sydney, NSW: NSW Government,; 2019.

29. NSW Treasury. NSW Gateway Policy. In: The Treasury, editor. Sydney, NSW: NSW Government,; 2017.

30. NSW Treasury. NSW Government Business Case Guidelines In: The Treasury, editor. Sydney, NSW: NSW Government,; 2018.

31. Infrastructure NSW. Infrastructure Investor Assurance Framework: Gateway Coordination Agency Framework for Capital Projects under the NSW Gateway Policy In: Infrastructure NSW, editor. Sydney: NSW Government; 2016.
